# Occurrence and Levels of Aflatoxins in Fish Feeds and Their Potential Effects on Fish in Nyeri, Kenya

**DOI:** 10.3390/toxins10120543

**Published:** 2018-12-17

**Authors:** Evalyn Wanjiru Mwihia, Paul Gichohi Mbuthia, Gunnar Sundstøl Eriksen, James K. Gathumbi, Joyce G. Maina, Stephen Mutoloki, Robert Maina Waruiru, Isaac Rumpel Mulei, Jan Ludvig Lyche

**Affiliations:** 1 Department of Veterinary Pathology, Microbiology and Parasitology, Faculty of Veterinary Medicine and Surgery, Egerton University, P.O. Box 536, Egerton 20115, Kenya; 2 Department of Food Safety and Infectious Biology, Faculty of Veterinary Medicine, Norwegian University of Life Sciences (NMBU), P.O. Box 8146, Oslo 0454, Norway; isaacmulei@yahoo.com; 3 Department of Pathology, Microbiology and Parasitology, Faculty of Veterinary Medicine, University of Nairobi, P.O. Box 29053, Kangemi 00625, Kenya; pgmbuthia@uonbi.ac.ke (P.G.M.); jkgathumbi@gmail.com (J.K.G.); rmwaruiru@uonbi.ac.ke (R.M.W.); 4 Toxinology Research Group, Norwegian Veterinary Institute, Ullevålsveien 68, Pb 750 Sentrum, Oslo 0106, Norway; gunnar.eriksen@vetinst.no; 5 Department of Animal Production, Faculty of Veterinary Medicine, University of Nairobi, P.O. Box 29053, Kangemi 00625, Kenya; maina.joyce78@gmail.com; 6 Department of Basic Sciences and Aquatic Medicine, Faculty of Veterinary Medicine, Norwegian University of Life Sciences (NMBU), P.O. Box 8146, Oslo 0454, Norway; stephen.mutoloki@nmbu.no

**Keywords:** aflatoxins, fish feed, Nyeri, Kenya, mycotoxins, ELISA

## Abstract

Aflatoxins are fungal metabolites that contaminate foods and feeds, causing adverse health effects in humans and animals. This study determined the occurrence of aflatoxins in fish feeds and their potential effects on fish. Eighty-one fish feeds were sampled from 70 farms and 8 feed manufacturing plants in Nyeri, Kenya for aflatoxin analysis using competitive enzyme-linked immunosorbent assay. Fish were sampled from 12 farms for gross and microscopic pathological examination. Eighty-four percent of feeds sampled tested positive for aflatoxins, ranging from 1.8 to 39.7 µg/kg with a mean of 7.0 ± 8.3 µg/kg and the median of 3.6 µg/kg. Fifteen feeds (18.5%) had aflatoxins above the maximum allowable level in Kenya of 10 µg/kg. Homemade and tilapia feeds had significantly higher aflatoxin levels than commercial and trout feeds. Feeds containing maize bran and fish meal had significantly higher aflatoxin levels than those without these ingredients. Five trout farms (41.7%) had fish with swollen abdomens, and enlarged livers with white or yellow nodules, which microscopically had large dark basophilic hepatic cells with hyperchromatic nuclei in irregular cords. In conclusion, aflatoxin contamination of fish feeds is prevalent in Nyeri, and may be the cause of adverse health effects in fish in this region.

## 1. Introduction

Fish farming in Kenya began in 1920 with the introduction of tilapia species, followed by common carp and African catfish [1]. Fish farming was inconsistent until 2009–2010 when the Government of Kenya invested in fish production through the Economic Stimulus Program (ESP). The aim of ESP was to stimulate economic development, alleviate poverty and promote food security and good nutrition [2,3]. The investment led to increased production of fish and fish products under aquaculture from approximately 1% in 2000–2004 to 8% in 2009–2011 [4] (p. 23). Aquaculture production in 2016 was estimated to be 14,960 metric tons [5].

Aquaculture development in Kenya is faced with several challenges such as unavailability of good quality and affordable fish feeds [6]. Fish feeds are the highest contributors to fish production costs and therefore greatly impact the economic returns from fish farming [7]. Additionally, fish nutrition and feed quality directly affect fish health and productivity. Feed quality is dependent on several factors such as raw materials used, processing conditions [8], nutritional value and feed management practices, among others. Together with low-quality feeds and feed ingredients, feed management practices such as poor storage, predispose fish feeds to contamination with aflatoxins [9].

Aflatoxins are highly toxic, carcinogenic, fungal, secondary metabolites produced mainly by the *Aspergillus* species [10]. These fungi are commonly found in most soils and they invade grains and other farm products used in animal feeds production, while they are still growing in the field (pre-harvest) or during storage (post-harvest) and produce aflatoxins when conditions are favorable [11]. There are at least 13 different types of aflatoxins but aflatoxins B_1_, B_2_, G_1_, and G_2_ are of most importance [12] with B_1_ considered most toxic and most prevalent [13]. The ubiquitous nature of *Aspergillus* fungi in soil makes it impossible to completely eliminate their invasion and subsequent aflatoxin production in most plant-based food/feedstuff.

Crops mostly affected by aflatoxin contamination include maize, groundnuts and cotton, but any feed crop that is stored is vulnerable [14]. Therefore, the use of products from cereals, oil seeds, groundnuts and cotton seeds in animal feeds, including fish feeds, may predispose animals to aflatoxin exposure and subsequent adverse health effects.

Depending on the exposure, contamination of fish feeds with aflatoxins can induce adverse health effects such as poor growth rates and presence of gross and microscopic lesions in fish. These lead to economic losses due to low production, morbidities, mortalities and poor quality of fish and fish products [15]. Exposure to highly contaminated feeds causes acute aflatoxicosis in fish characterized by pale gills, impaired blood clotting, anaemia, poor growth rates and death. Chronic exposure through prolonged feeding of lower aflatoxin concentrations causes tumors in livers and kidneys of fish [16].

Aflatoxin contamination of feeds is a worldwide problem. Fallah et al. [17], Barbosa et al. [18], Rodríguez-Cervantes et al. [19] and Dutta and Das [20] reported presence of *Aspergillus* fungi and over 50% occurrence of aflatoxins in fish feeds in Iran, Brazil, Mexico and India, respectively. In East Africa, only Marijani et al. [21] reported a 64.3% occurrence of aflatoxins in fish feeds with levels of up to 806.9 µg/kg in feeds from the Lake Victoria region in Kenya. There is therefore little knowledge on occurrence, levels of aflatoxins in fish feeds and their potential effects on fish in this region. The aims of this study were twofold: (1) to determine the occurrences and levels of aflatoxins in fish feeds and (2) to determine whether aflatoxins in the feeds are associated with adverse fish health effects in Nyeri County, Kenya.

## 2. Results

### 2.1. Fish Feed Analysis

A total of 204 fish farmers and 8 fish feed manufacturers in Nyeri were visited. Twenty-three farmers (11.3%) acknowledged feeding their fish exclusively on leafy vegetables from their farms while 181 farmers fed their fish with commercial and/or homemade feeds. Of the 181 farmers, only 70 farmers (38.7%) were in possession of fish feeds at the time of sampling. In total, 81 fish feed samples were collected from the fish farms and fish feed manufacturing plants.

Sixty-eight feeds (84.0%) tested positive for total aflatoxins ranging from the enzyme-linked immunosorbent assay (ELISA) kit’s limit of detection (LOD) of 1.8 to 39.7 µg/kg while 13 samples (16.0%) had aflatoxin levels below the LOD of 1.8 µg/kg. The mean total aflatoxins level was 7.0 ± 8.3 µg/kg (95% confidence interval [CI], 5.2–8.8 µg/kg) and the median level was 3.6 µg/kg (95% CI, 2.9–4.5 µg/kg). Further analysis using liquid chromatography–high-resolution mass spectrometry (LC-HRMS/MS) indicated that aflatoxins B_1_ and G_1_ were present in the fish feeds (data not shown). Aflatoxins B_2_ and G_2_ were not detected in any of the samples tested.

**Figure 1:**
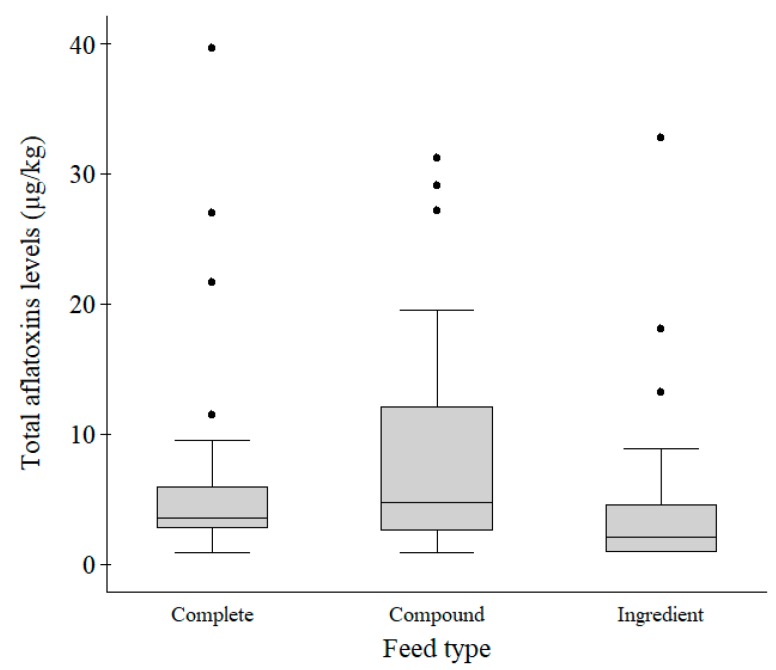
Box plot comparing total aflatoxins levels in complete (*n* = 37), compound (*n* = 26) and ingredient (*n* = 18) feed types. The aflatoxins levels were not significantly different (*p* = 0.10) among the feed types. Legend: The grey boxes represent the middle 50% of the data in the group. The lines through the boxes represent the medians. The bottom and top of each box represent the 25th and 75th percentiles, respectively. The lines (whiskers) extending from the box represent 10th and 90th percentiles, respectively. The black dots represent individual outliers.

Feed material/ingredient mixtures of which composition contained all nutrients sufficient for a daily ration were considered as a complete feed, whereas feed mixtures of which composition did not have all nutrients were taken as a compound feed [22]. Under this categorization, the 81 samples were comprised of 37 (45.7%) complete feeds, 26 (32.1%) compound feeds and 18 (22.2%) ingredients (Table 1 and Figure 1). The Kruskal–Wallis test showed that total aflatoxins levels were not significantly different (χ^2^ = 4.58; *p* = 0.10) among complete, compound and ingredients feed types.

Five (13.5%) complete and nine (34.6%) compound feeds had aflatoxins levels above 10 µg/kg which is the maximum level (ML) for aflatoxins allowed in both complete and compound animal feeds [22,23] (Table 1 and Figure 1). Only one (5.6%) ingredient had an aflatoxin level above 20 µg/kg which is the ML for aflatoxins allowed in feed ingredients [22]. In total, 15 (18.5%) samples had aflatoxins levels above the ML set by Kenya Bureau of Standards (KEBS) and the European Commission.

**Figure 2:**
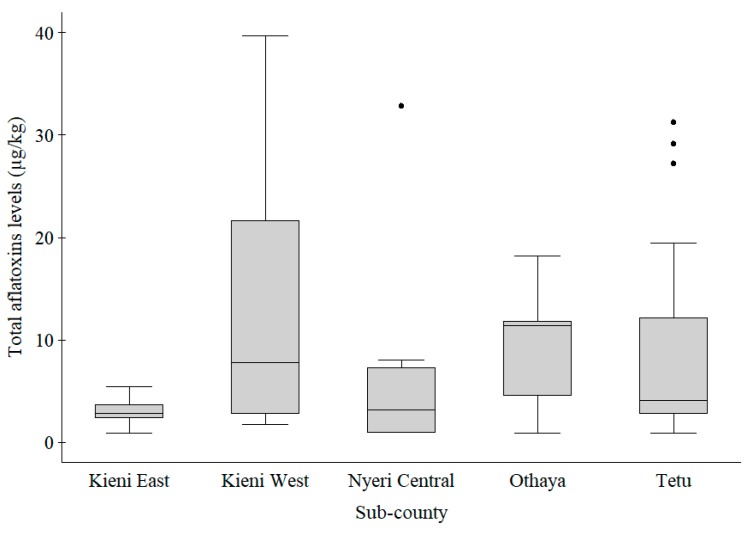
Box plot comparing total aflatoxins levels in feed samples from 5 sub-counties in Nyeri County. The aflatoxins levels were significantly different (*p* = 0.01) among the sub-counties. Kieni East had significantly lower levels than Tetu (*p* = 0.01), Othaya (*p* = 0.00) and Kieni West (*p* = 0.04). Legend: The grey boxes represent the middle 50% of the data in the group. The lines through the boxes represent the medians. The bottom and top of each box represent the 25th and 75th percentiles, respectively. The lines (whiskers) extending from the box represent 10th and 90th percentiles, respectively. The black dots represent individual outliers.

Fish feed samples from Tetu, Kieni East, Nyeri Central, Kieni West and Othaya constituted 37.0%, 29.6%, 13.6%, 11.1% and 8.6% of all the samples collected, respectively (Table 2 and Figure 2). Kruskal–Wallis test showed that total aflatoxins levels were significantly different (χ^2^ = 12.56; *p* = 0.01) among the five sub-counties. Total aflatoxins levels in feeds from Othaya, Tetu and Kieni West were not significantly different from each other. However, Kieni East had a significantly lower median than Tetu (χ^2^ = 7.05; *p* = 0.01), Othaya (χ^2^ = 7.90; *p* = 0.00) and Kieni West (χ^2^ = 4.48; *p* = 0.04). Nyeri Central had a significantly lower (χ^2^ = 3.79; *p* = 0.05) median than Othaya. On Fisher’s test, the sub-counties sampled were found to be associated with the fish reared (*p* < 0.01), feed groups (*p* = 0.001), feed types (*p* < 0.01), feed forms (*p* = 0.001) and feed sources (*p* = 0.003). Out of the 15 samples with total aflatoxins levels above the maximum allowable level, 7 (46.7%) samples were from Tetu with 4 (26.7%) from Othaya, 3 (20.0%) from Kieni West, 1 (6.7%) from Nyeri Central and none (0.0%) from Kieni East.

Data on total aflatoxins levels in fish feed samples by feed characteristics are shown in Table 3.

Seventy (86.4%) fish feeds were collected from fish farms while 11 (13.6%) were from fish feed manufacturing plants (Table 3). The Mann–Whitney test showed that total aflatoxins levels were not significantly different (z = 0.32; *p* = 0.75) between feeds from fish farms and feed manufacturing plants.

**Figure 3:**
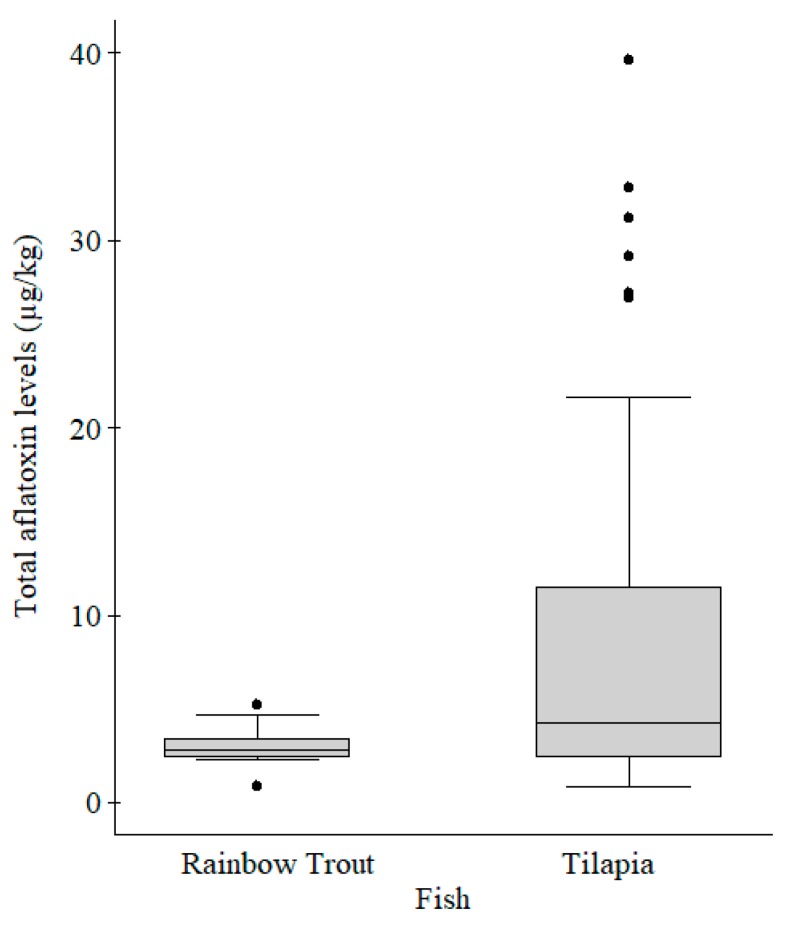
Box plot comparing total aflatoxins levels in rainbow trout (*n* = 17) and tilapia (*n* = 64) fish feed samples. The aflatoxins levels were significantly different (*p* = 0.03) between rainbow trout and tilapia feeds. Legend: The grey boxes represent the middle 50% of the data in the group. The lines through the boxes represent the medians. The bottom and top of each box represent the 25th and 75th percentiles, respectively. The lines (whiskers) extending from the box represent 10th and 90th percentiles, respectively. The black dots represent individual outliers.

Majority of the feeds (79.0%) were for tilapia while 21.0% were for rainbow trout (Table 3). The Mann–Whitney test showed that total aflatoxins median level was significantly higher (z = −2.13, *p* = 0.03) in tilapia feeds than in rainbow trout feeds (Figure 3). On Fisher’s test, the fish reared were found to be associated with feed types (*p* < 0.01), feed groups (*p* < 0.01), feed forms (*p* < 0.01) and feed sources (*p* < 0.01). All samples with total aflatoxins levels above the maximum allowable level were tilapia feeds, constituting 30.6% of all the tilapia feeds tested.

**Figure 4:**
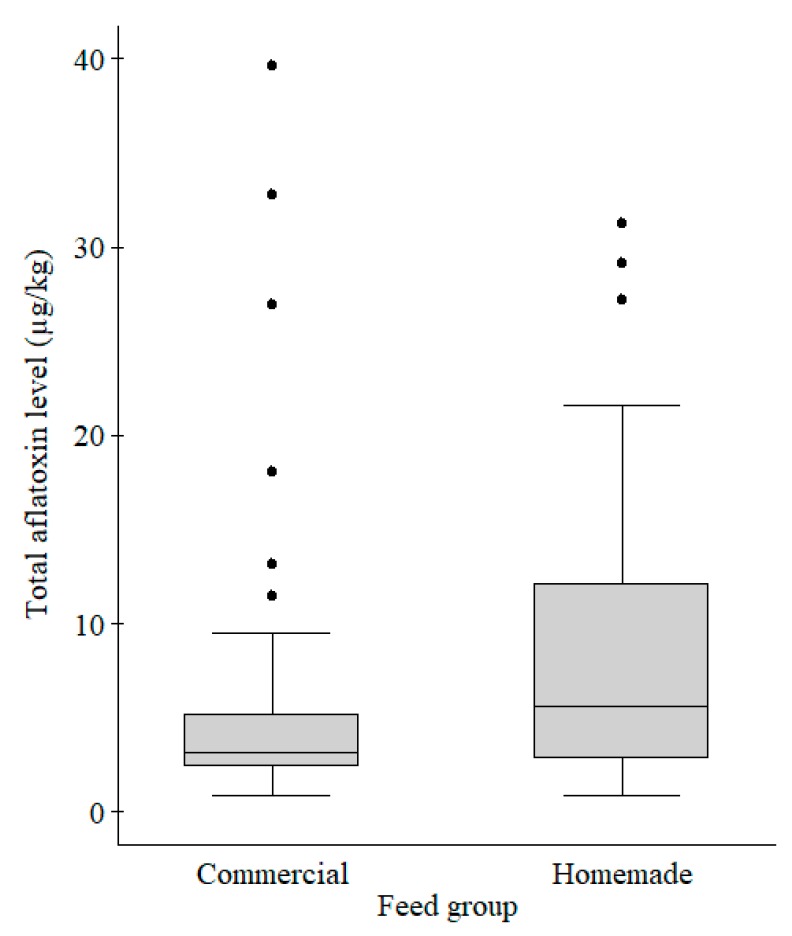
Box plot comparing total aflatoxins levels in commercial (*n* = 51) and homemade (*n* = 30) fish feed samples. The aflatoxins levels were significantly different (*p* = 0.05) between commercial and homemade feeds. Legend: The grey boxes represent the middle 50% of the data in the group. The lines through the boxes represent the medians. The bottom and top of each box represent the 25th and 75th percentiles, respectively. The lines (whiskers) extending from the box represent 10th and 90th percentiles, respectively. The black dots represent individual outliers.

The feed samples were either commercial (63.0%) or homemade (37.0%) (Table 3). The Mann–Whitney test showed that total aflatoxins median level was significantly higher (z = −1.96, *p* = 0.05) in homemade than in commercial feeds (Figure 4). Of the 15 samples that had total aflatoxins values above the maximum allowable level, 11 (73.3%) were homemade, constituting 36.7% of all homemade feeds.

The feed samples were mostly mash (49.4%) or pellet (37.0%) in form (Table 3). The Kruskal–Wallis test showed that total aflatoxins levels were not significantly different (χ^2^ = 6.38; *p* = 0.17) among the different forms of the fish feeds.

Ingredients were analyzed for 55 (67.9%) fish feeds collected as shown in Table 4 and Table 5. Forty-six feeds (83.6%) contained cereal milling by-products; 23 (41.8%) contained animal proteins; 10 (18.2%) contained oilseed cakes or meal and 7 (12.7%) contained cereal grains. Total aflatoxins levels in feeds containing the four ingredient groups were not significantly different (χ^2^ = 2.17; *p* = 0.54) from each other.

The top six ingredients mostly used for preparation of fish feeds were wheat bran (52.7%), maize bran (45.5%), pollard (25.5%), dried silver cyprinid fish (16.4%), fish meal (16.4%) and cotton seed cake (12.7%). Of the 15 feed samples with total aflatoxins levels above the maximum allowable level, the majority contained maize bran (8, 53.3%) and wheat bran (5, 33.3%) (Table 5). The Mann–Whitney test showed that total aflatoxins median levels were significantly higher in feeds containing either maize bran (z = −2.43; *p* = 0.01) or fish meal (z = −2.59; *p* = 0.01) than those without these two ingredients. Fifty-two point one percent (52.1%) and 18.8% of tilapia feeds contained maize bran and fish meal, respectively, whereas none of the rainbow trout feeds had these ingredients. Similarly, 74.1% and 29.6% of homemade feeds contained maize bran and fish meal, respectively, but only 17.9% and 3.6% of the commercial feeds had these ingredients.

### 2.2. Fish Health Problems Reported

Twenty-two (10.8%) fish farms visited reported fish health problems. Of these, 31.8%, 9.1%, 45.5% and 36.4% reported fish mortalities, poor appetites, poor growth rates and tumor-like lesions in fish, respectively (Table 6). Fisher’s exact test showed that rainbow trout farms reported a significantly higher (*p* < 0.001) occurrence of tumor-like lesions in their fish (87.5%) than tilapia farms (7.1%) (Table 6). However, reports of mortalities, poor feed intake and growth rates were not significantly different between tilapia and rainbow trout farms.

### 2.3. Fish Examination

A total of 120 fish, 10 fish from each of the 12 farms sampled, were examined grossly and microscopically for lesions. Eighty of the 120 (66.7%) fish examined were rainbow trout while the remaining were tilapia. Rainbow trout (57.5%) showed significantly more (*p* < 0.001) gross and microscopic lesions than tilapia fish (5.4%).

**Figure 5:**
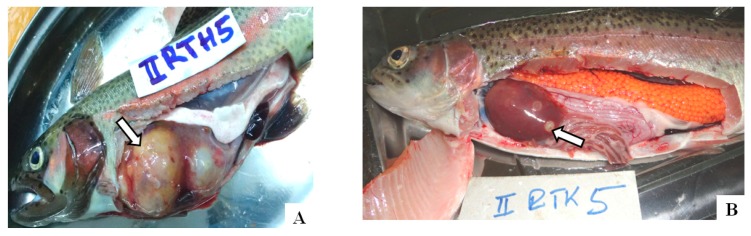
(**A**,**B**) Rainbow trouts with multiple, yellow-grey nodular swellings (white arrow) in the livers.

Post-mortem examination of rainbow trouts sampled from the study farms showed swollen abdomens with ascites (46.3%) and markedly enlarged livers (60.0%) with single or multiple whitish or yellow nodules or cystic swellings (50.0%) (Figure 5). The majority of the trout livers had areas of necrosis (61.3%) and haemorrhages (91.3%). Muscular haemorrhages (30.0%) and enlarged hearts (40.0%) and kidneys (56.3%) were also observed (Table 7). Rainbow trouts (50.8%) showed significantly more (*p* < 0.001) gross lesions than tilapia fish (5.9%).

**Figure 6:**
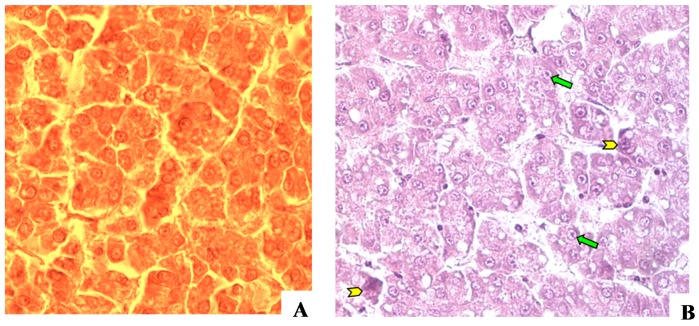
Rainbow trout liver sections showing (**A**) normal hepatocytes organized into regular cords and (**B**) abnormal, hyperchromatic hepatocytes (yellow arrowheads) forming thick, irregular cords and containing large nuclei (green arrows) with prominent nucleolus (Haematoxylin and Eosin [H&E], ×400).

Histological examination of over 45.0% of trout livers showed various degrees of irregular cords of dark, large, basophilic, abnormal hepatocytes with large, hyperchromatic nuclei with prominent nucleolus (Figure 6). These findings are suggestive of hepatomas which are usually associated with aflatoxin exposure. Rainbow trout (66.5%) showed significantly more (*p* < 0.001) microscopic lesions than tilapia fish (4.6%), most of which were suggestive of hepatomas.

Total aflatoxins levels in feeds sampled from tilapia farms (median = 10.5 µg/kg) were significantly higher (*p* = 0.01) than feeds from rainbow trout farms (median = 2.8 µg/kg). However, no significant difference (*p* = 0.89) in total aflatoxins levels was detected in feeds from farms with fish showing pathological lesions (median = 2.8 µg/kg) and those that were not showing lesions (median = 2.8 µg/kg).

Three trout farms (37.5%) and 1 tilapia farm (25.0%) sampled manufactured their own fish feeds in-house, whereas the remaining farms sourced their fish feeds from different sources including the farms that manufactured their own feeds. No significant difference (*p* = 0.73) in total aflatoxins levels was, however, detected in feeds from farms that manufactured their own feeds (median = 2.8 µg/kg) and those that purchased the feeds from outside sources (median = 3.2 µg/kg).

## 3. Discussion

This study confirmed presence of aflatoxins in fish feeds for tilapia and rainbow trout in Nyeri County, Kenya. Aflatoxin occurrence in fish feeds was found to be higher (84.0%) than that reported by Marijani et al. [21], who detected aflatoxins in 16 (36.5%) fish feeds from the Lake Vitoria area in Kisumu, Kenya. However, Marijani et al. reported higher levels of 90.1, 9.9 and 22.1 µg/kg of aflatoxin B_1_, B_2_ and G_1,_ respectively, than those shown in the present study. These authors analyzed tilapia feeds, but not trout feeds. The higher levels of aflatoxins reported could be due to warmer weather in Kisumu County compared to those in Nyeri County. Marijani et al. reported an average temperature of 32 °C during sample collection which is higher than that recorded in the present study that ranged between 16.3 °C and 18.8 °C. Relative humidity recorded in the present study, 73.8–84.9%, was similar to 78% recorded by Marijani et al. Fallah et al. [17] reported a lower occurrence of total aflatoxins (67.4%) in fish feeds in Iran with a wider range of 0.5–68.5 µg/kg. Similarly, Dutta and Das [20] reported a lower occurrence of 76.2% in fish feeds in India with a very high mean of 412 ± 154 µg/kg. Marijani et al. and Dutta and Das attributed the higher aflatoxins levels to high ambient temperature and relative humidity, together with inappropriate feed handling and storage practices. Other predisposing factors for aflatoxin contamination include type of ingredient, moisture content, damage by insects/rodents [16], soil type, water activity, harvest time, drying time [24], among others, which were neither evaluated in the present study nor discussed by Marijani et al., Fallah et al. and Dutta and Das.

Aflatoxins, and mycotoxins in general, are difficult to completely avoid in food and feed products, therefore, maximum levels (ML) are set to assure food and feed safety [25]. The MLs set by the Kenya Bureau of Standards and the European Commission for complete and compound animal feeds are 10 µg/kg [22,23] and 20 µg/kg for feed ingredients [22]. Exposure of fish to low doses of aflatoxins for a long period of time [16] may lead to chronic aflatoxicosis and a risk of aflatoxin residue accumulation in fish tissue [26,27]. Michelin et al. [27] have shown accumulation of aflatoxins is lambari (*Astyanax altiparanae*) fish liver and muscle after 90 days of exposure. Consumption of fish containing aflatoxin residues may cause adverse health effects ranging from acute hepatic toxicity to chronic disease, such as liver cancer, haemorrhages, oedema, and even immediate death in humans [28].

In fish, aflatoxicosis has been associated with adverse health effects such as impaired blood clotting, immune suppression, poor growth rates, reduced appetites, hepatic carcinomas and mortalities [9,29]. Over 10% of fish farmers in Nyeri County reported cases of poor growth rates, poor appetite, mortalities and tumors, which could be attributable to aflatoxin exposure through contaminated fish feeds. Once aflatoxin-contaminated feed is consumed, aflatoxins are absorbed from the ingesta and passed to different organs. The principal target organ for aflatoxins is the liver [30]. After the invasion of aflatoxins into the liver, lipids infiltrate hepatocytes, which leads to necrosis or liver cell death [9]. Aflatoxins bind to DNA, creating the aflatoxin B_1_ exo-8,9-epoxide which is involved in the development of fatty liver, necrosis and carcinogenesis in fish and other animals.

Rainbow trouts are very sensitive to aflatoxins [31] with a median lethal dose (LD_50_) of less than 1000 µg/kg body weight [16]. Sensitivity also varies with age, and fry are more vulnerable than adult fish [16]. Tilapia are less susceptible to aflatoxicosis than rainbow trout [32]. Levels as low as 0.01 µg/kg of aflatoxin have been reported to induce neoplastic changes in rainbow trout over a relatively short period [33] (p. 419). It has been shown that prolonged feeding of 3–6 months with doses of 1–20 µg/kg [34] of aflatoxin, which are similar to or lower than the levels measured in the present study (1.8–29.7 µg/kg), induced liver tumors (malignant hepatocellular carcinomas) in rainbow trout [35,36]. In their study, Anh-Tuan et al. [32] showed that acute and sub-chronic effects of aflatoxins to Nile tilapia are unlikely if dietary concentrations are 250 µg/kg or less. A higher percentage of swellings and tumors were diagnosed in the rainbow trout farms compared to that in the tilapia farms, possibly because of greater sensitivity of rainbow trout to aflatoxins than tilapia. Additionally, rainbow trout farmers feed their fish exclusively on commercial feeds while the tilapia farmers feed their fish on combinations of commercial feeds, homemade feeds and leafy vegetables from their farms. Some tilapia farmers admitted that they did not feed their fish daily. This means that the level and rate of exposure of tilapia to aflatoxin-contaminated feeds was less than that of rainbow trout even though the dose within the tilapia feeds was higher than that in trout feeds. Experiments by Deng et al. [35] indicated that aflatoxicosis in tilapia depended on both dose and duration, which may explain the fewer lesions observed in tilapia fish in the present study.

Selective sensitivity towards aflatoxins in fish, for example between rainbow trout and tilapia, is due to differences in the pattern of enzymes involved in aflatoxin metabolism. Such differences might be ascribed to a different gene expression or enzyme efficiency, and consequently to an altered balance in the aflatoxin metabolic pathway [36]. In fish, as in mammals, the metabolic pathway of aflatoxins is characterized by two routes with two major catalysts systems: the activation phase mediated by cytochrome P450-dependent mixed-function oxidases, and the detoxification phase comprised of the two most important detoxifiers, the uridine diphosphate (UDP) glucuronyl-transferase (UDPGT) and glutathione (GSH)-S-transferase (GST) [37]. Rainbow trout sequestrate aflatoxins via a highly efficient microsomal epoxidation, thereby activating the aflatoxins to aflatoxin B_1_ exo-8,9-epoxide [37] and expressing little GST activity towards the aflatoxin B_1_ exo-8,9-epoxide [38], leading to its high sensitivity to aflatoxins. Additionally, the high responsiveness of rainbow trout to cancer induction might be also related to the poor efficiency of its DNA repair system in removing bulky adducts [39]. Resistant species like Coho salmon, channel catfish and tilapia are less sensitive to aflatoxins because they poorly oxidize aflatoxins and rapidly convert aflatoxins to aflatoxicol that allows for rapid elimination of free aflatoxins [37].

In order to assess whether disease conditions related to aflatoxin exposure occurred in the farms studied, fish were collected for pathological examination. Post-mortem examination showed tumor-like lesions in 5 of 8 (62.5%) trout farms, affecting 50% of the trout. However, no tumor-like lesions were detected in tilapia. Histopathological examination of the rainbow trout livers showed irregular cords with abnormal, basophilic hepatocytes containing large nuclei and prominent nucleoli, which were consistent with aflatoxin-induced hepatomas. This is congruent with studies by Rajeev-Raghavan et al. [40], Mahfouz and Sherif [15], Arana et al. [41], Zychowski et al. [30] and Shahafve et al. [42], who reported similar findings in sturgeon, Nile tilapia, rainbow trout, red drum and common carp exposed to various levels of aflatoxins. However, no tumors or histological changes consistent with aflatoxin-induced hepatomas were detected in tilapia, probably because of the above-mentioned greater sensitivity of rainbow trout to aflatoxins compared with tilapia [32,37].

There was no significant difference (*p* = 0.89) in total aflatoxins levels detected in feeds from farms with fish showing pathological lesions (median = 2.8 µg/kg) and those that were not showing lesions (median = 2.8 µg/kg). It is a challenge to directly attribute the pathological lesions observed at post-mortem examination with the levels of total aflatoxins measured, because the feed from the farms with pathological lesions were only analyzed at one point in time (cross-sectional design) and the lesions seen are associated with a chronic disease condition which takes time to develop [43]. This means that induction of the lesions may have been caused by earlier feed batches with unknown aflatoxin levels, rather than by the feed they had at the farm during the investigation. In acute cases of aflatoxicosis, the levels of aflatoxins identified in feed are usually higher [44] (p. 49) than those observed in this study. Prospective cohort studies are therefore suggested in Nyeri County to adequately associate the pathological lesions observed in the fish to the levels of aflatoxins in the feeds fed.

Feeds from all sub-counties except Nyeri Central had significantly higher median total aflatoxins levels than those in Kieni East. All rainbow trout farms visited were located in Kieni East sub-county where ambient and water temperatures are lower than 15 °C which is ideal for rearing this type of fish (6–20 °C) [34]. The feeds from Kieni East sub-county were produced and stored at lower temperatures which are suboptimal for aflatoxin production [16]. This explains the association found between sub-counties and fish reared. Rainbow trout were fed exclusively on commercial feeds which were mainly complete and pelleted, therefore accounting for the associations found between fish reared and feed groups, feed types and feed sources. Tilapia, on the other hand, tended to be fed on homemade feeds, compound feeds or ingredients which were mostly in mash form.

In this study, fish feeds containing maize bran, wheat bran, fish meal, cotton seed cake, sunflower seed cake and soya meal contained aflatoxin levels above maximum allowable limits. Maize, cotton seed, sunflower seed, soya bean and groundnuts have been reported to be commonly contaminated with aflatoxins in Africa due to the tropical climate [35]. In Kenya, there are at least four reports of large-scale aflatoxin contamination of maize between 2004 and 2016 [2,45,46,47,48]. Significantly high aflatoxin levels were detected in feeds with maize bran and fish meal. Similar findings by Alinezhad et al. [49] showed significantly high levels of aflatoxins in fish meal (mean = 67.4 µg/kg) compared to wheat (mean = 12.4 µg/kg), wheat flour (mean = 2.3 µg/kg) and starch (mean = 1.8 µg/kg) used to feed fish in Iran [49]. Higher proportions of tilapia feeds and homemade feeds contained maize bran and fish meal compared to those of rainbow trout feed and commercial feeds, possibly explaining why they had significantly higher levels of aflatoxins.

## 4. Conclusions

This study has shown that fish feeds used in Nyeri county were contaminated with aflatoxins and that the rainbow trout in this region showed lesions typical of aflatoxin-induced hepatomas, indicating that they might have been exposed to aflatoxins at some point in their life. The occurrences and levels of aflatoxins in the feeds sampled indicated a major problem in controlling invasion of feeds and feed ingredients with *Aspergillus* fungi and aflatoxins. The fish production problems reported by fish farmers in Nyeri could potentially be due to aflatoxin exposure, which directly affects fish health and predisposes them to other diseases and cancers. Strategies to control aflatoxin exposure and its effects need to be implemented to prevent losses in fish production industry. Fish feeds need to be monitored to ensure that the feeds have aflatoxins levels below maximum allowable levels, thus safeguarding fish health. Similarly, aflatoxins residues in the fish need to be monitored to ensure that fish and fish by-products are safe for human consumption.

## 5. Materials and Methods

### 5.1. Study Area

This was a cross-sectional study carried out between August and December 2015 in Kieni East, Kieni West, Nyeri Central, Tetu and Othaya sub-counties of Nyeri County. Nyeri County covers an area of 3337.1 km^2^ [50]. It lies between longitude 36°–38° East and latitude 0° 0′–0° 38′ South [51] with altitudes of between 3076–5199 m above the sea level [52]. Temperatures range between 12.8 °C and 20.8 °C and annual rainfalls vary from 500 to 1600 mm. The natural water towers, Aberdare mountain ranges in the West and Mount Kenya in the East, form part of Nyeri County borders and provide cold water for rainbow trout farming.

### 5.2. Feed Sample Collection

Fish feeds were sampled from fish farms and fish feed manufacturing plants. Representative samples in at least 5 increments totaling to 1 kg each were collected from each feed and/or ingredient package from the top, middle and bottom of the package as per KS ISO 6497:2002 standard on animal feeding stuff sampling [53]. The feed samples were packed in paper bags, wrapped in polythene bags and allocated with unique identification numbers. Feed samples data recorded included feed type, source, form, ingredients and type of fish to be fed. The feed samples were transported to the laboratory and stored in a freezer at −20 °C until analyzed at the Department of Public Health, Pharmacology and Toxicology at the University of Nairobi.

### 5.3. Feed Sample Preparation and Testing

The samples were prepared and tested for total aflatoxins levels using a competitive total aflatoxins enzyme-linked immunosorbent assay (ELISA) kit (Ridascreen^®^ Aflatoxin Total, R4701, R-Biopharm AG, Darmstadt, Germany) as per manufacturer’s instructions [54]. Each feed sample was brought to room temperature, mixed thoroughly and ground to a fine powder using a knife mill (Retsch Grandomix GM200, Haan, Germany) at 7500 rpm for 1 min. Two grams of the ground feed were weighed into a 50 mL plastic tube (Falcon, Thermo Fisher Scientific, Waltham, MA, USA) and 10 mL of methanol: distilled water (70:30, *v*/*v*) mixture was added. Mixing was done at room temperature using a shaker (Burrell wrist action shaker, model 75, Pittsburgh, PA, USA) for 10 min. The mixture was filtered through Whatman^®^ filter paper No.1 (GE Healthcare Bio-Sciences, Pittsburgh, PA, USA) with an 11 µm pore size and 100 µL of the filtrate was diluted with 600 µL distilled water. Fifty microliters of the filtered sample extracts and standard dilutions were pipetted into the microtiter plates in duplicate wells. Fifty microliters of peroxidase-conjugated aflatoxin B_1_ followed by 50 μL of the monoclonal anti-aflatoxin antibodies solution were added into each well. The plate was incubated for 30 min in a shaking incubator (Thermo Scientific 4625Q 4-Place Microplate Shaker, Waltham, MA, USA) at room temperature. The wells were emptied and cleaned twice using 250 μL of washing buffer. A hundred microliters of substrate were added to each well and incubated for 15 min at room temperature. A stop solution of 100 μL was added to each well and absorbance was measured at 450 nm using an ELISA reader (Thermo Electron Corporation Multiskan EX, Waltham, MA, USA). Total aflatoxins levels were calculated using ELISA software (Rida^®^Soft, Z9999, R-Biopharm AG, Darmstadt, Germany). Aflatoxin levels below the kit’s limit of detection (LOD) of 1.8 µg/kg were assigned half the LOD value of 0.9 µg/kg in the statistical calculations. The documented ELISA kit’s recovery rate was approximately 85% with cross reactivity of 100% for Aflatoxin B_1_, with 48% for Aflatoxin B_2_, 75% for Aflatoxin G_1_ and 18% for Aflatoxin G_2_ [54].

### 5.4. Fish Sample Collection and Examination

Twelve farms, eight rearing rainbow trout and four rearing tilapia were selected for fish sampling. Ten fish per farm were sampled and post-mortem examination was carried out. The fish and their organs were examined for gross pathological lesions. Livers from the fish were fixed in 10% buffered formalin for histological processing, as described by Brar et al. [55]. The 3–5 µm thick haematoxylin and eosin stained liver tissues mounted on slides with cover slips were examined for microscopic lesions.

### 5.5. Data Analysis

Feed data on type, source, form, ingredients and aflatoxin levels were entered into Microsoft^®^ Excel 2007 (Microsoft Corporation, Redmond, WA, USA) spreadsheets, checked and corrected for transcription errors. They were exported into Stata/SE 14.2 (StataCorp LLC, College station, TX, USA) for statistical analysis. Summary statistics were generated and numeric variables expressed as median, and mean ± standard deviation with the 95% confidence interval reported. The data were skewed to the right and therefore non-parametric tests such as Mann–Whitney and Kruskal–Wallis tests were used to compare medians. Significant observations were reported at *p* ≤ 0.05.

## Figures and Tables

**Table 1:** Total aflatoxins levels in complete, compound and ingredient feeds.

Feed Type	Occurrence	≥ML	Range	Median	Median 95% CI	Mean ± SD	Mean 95% CI
(*n* = 81)	% (*n*)	% (*n*)	µg/kg	µg/kg	µg/kg	µg/kg	µg/kg
Complete	45.7 (37)	13.5 (5)	<1.8–39.7	3.6	2.9–4.7	6.7 ± 7.7	3.6–8.9
Compound	32.1 (26)	34.6 (9)	<1.8–31.2	4.8	2.8–12.0	8.9 ± 9.2	5.3–12.4
Ingredient	22.2 (18)	5.6 (1)	<1.8–32.8	2.1	0.9–4.4	5.6 ± 8.3	1.7–9.5

Key: ≥, greater than or equal to; ML, maximum level; CI, confidence interval; SD, standard deviation; µg/kg, micrograms per kilogram; <, less than.

**Table 2:** Total aflatoxins levels in fish feed samples from each sub-county.

Sub-County (*n* = 81)	Occurrence % (*n*)	≥ML % (*n*)	Range µg/kg	Median µg/kg	Median 95% CI µg/kg	Mean ± SD µg/kg	Mean 95% CI µg/kg
Tetu	37.0 (30)	23.3 (7)	<1.8–31.2	4.1 *	3.0–6.9	8.3 ± 8.6	5.1–11.4
Kieni East	29.6 (24)	0.0 (0)	<1.8–5.5	2.8	2.4–3.6	2.9 ± 1.3	2.3–3.4
Nyeri Central	13.6 (11)	11.1 (1)	<1.8–32.8	3.2	0.9–7.5	5.9 ± 9.3	0.3–11.5
Kieni West	11.1 (9)	33.3 (3)	1.76–39.7	7.8 *	2.0–26.5	12.9 ± 13.4	4.0–21.8
Othaya	8.6 (7)	57.1 (4)	<1.8–18.2	11.4 *	2.0–16.2	9.9 ± 5.6	5.5–13.9

Key: ≥, greater than or equal to; ML, maximum level; CI, confidence interval; SD, standard deviation; µg/kg, micrograms per kilogram; <, less than. * Othaya (*p* = 0.00), Tetu (*p* = 0.10) and Kieni West (*p* = 0.04) median levels significantly higher than Kieni East.

**Table 3:** Total aflatoxins levels in fish feed samples categorized by feed characteristic.

Characteristics		Occurrence	≥ML	Range	Median	Median 95% CI	Mean ± SD	Mean 95% CI
		% (*n*)	% (*n*)	µg/kg	µg/kg	µg/kg	µg/kg	µg/kg
**Source of fish feed (*n* = 81)**								
	Fish farmers	86.4 (70)	18.6 (13)	<1.8–39.7	3.8	3.1–4.7	7.2 ± 8.1	5.1–9.3
	Manufacturer	13.6 (11)	18.2 (2)	2.34–18.2	2.8	2.4–10.0	5.6 ± 5.2	2.5–8.7
**Type of fish fed (*n* = 81)**								
	Rainbow trout	21.0 (17)	0.0 (0)	<1.8–5.2	2.8	2.4–3.4	3.0 ± 1.0	2.5–3.4
	Tilapia	79.0 (64)	23.4 (15)	<1.8–39.7	4.0 ^a^	3.2–6.0	8.1 ± 9.1	5.8–10.3
**Feed group (*n* = 81)**								
	Commercial	63.0 (51)	7.8 (4)	<1.8–39.7	3.2	2.8–4.0	5.7 ± 7.8	3.6–7.9
	Homemade	37.0 (30)	36.7 (11)	<1.8–31.2	5.6 ^b^	3.2–11.8	9.2 ± 8.9	5.9–12.4
**Form of feed (*n* = 81)**								
	Pellets	37.0 (30)	10.0 (3)	<1.8–39.7	3.2	2.8–4.6	5.9 ± 7.9	3.0–8.8
	Crumble	4.9 (4)	0.0 (0)	<1.8–5.2	3	0.9–5.3	3.0 ± 2.0	1.1–5.0
	Mash	49.4 (40)	30.0 (12)	<1.8–32.8	4.3	3.2–10.7	8.9 ± 9.2	6.1–11.8
	Fine/Flour	6.2 (5)	0.0 (0)	<1.8–7.8	0.9	0.9–7.8	2.9 ± 3.1	0.1–5.6
	Cake	2.5 (2)	0.0 (0)	<1.8–3.2	2	0.9–3.2	2.0 ± 1.6	−4.6

Key: ML, maximum level; CI, confidence interval; SD, standard deviation; ≥, greater than or equal to; <, less than; µg/kg, micrograms per kilogram. ^a^ Tilapia feeds total aflatoxins levels significantly higher (*p* = 0.03) than levels in trout feeds. ^b^ Homemade feeds total aflatoxins levels significantly higher (*p* = 0.05) than levels in commercial feeds.

**Table 4:** Total aflatoxins levels in fish feed samples as per ingredient group.

Ingredient Group	Occurrence	≥ML	Range	Median	Median 95% CI	Mean ± SD	Mean 95% CI
(*n* = 55)	% (*n*)	% (*n*)	µg/kg	µg/kg	µg/kg	µg/kg	µg/kg
Cereal milling by-products	83.6 (46)	21.7 (10)	<1.8–32.8	3.5	2.8–5.6	7.6 ± 8.7	5.0–10.1
Animal proteins	41.8 (23)	21.7 (5)	<1.8–29.1	4.0	3.2–8.8	7.8 ± 7.4	4.7–11.0
Oilseed cakes or meal	18.2 (10)	40.0 (4)	<1.8–29.1	9.2	2.1–20.5	10.8 ± 9.4	4.8–16.8
Cereal grains	12.7 (7)	28.6 (2)	2.2–13.4	3.2	2.3–13.0	6.4 ± 4.9	2.6–10.1

Key: ML, maximum level; CI, confidence interval; SD, standard deviation; ≥, greater than or equal to; <, less than; µg/kg, micrograms per kilogram.

**Table 5:** Total aflatoxins levels in fish feeds categorized by ingredients contained in the feeds.

Ingredient	Occurrence	≥ML	Range	Median	Median 95% CI	Mean ± SD	Mean 95% CI
(*n* = 55)	% (*n*)	% (*n*)	µg/kg	µg/kg	µg/kg	µg/kg	µg/kg
**Oilseed cake or meal**							
Cotton seed cake	12.7 (7)	28.6 (2)	<1.8–29.1	8.9	1.2–23.6	9.4 ± 9.6	2.1–16.6
Sunflower seed cake	9.1 (5)	60.0 (3)	2.7–21.6	11.5	2.7–21.6	12.7 ± 7.4	6.0–19.3
Canola cake	1.8 (1)	100.0 (1)	18.2	18.2	-	18.2	-
Soya bean meal	5.5 (3)	66.7 (2)	9.48–21.6	3.3	11.5–9.5	14.2 ± 6.5	6.7–21.7
**Cereal milling by-products**							
Wheat bran	52.7 (29)	17.2 (5)	<1.8–32.8	3.7	2.8–6.0	7.8 ± 9.0	4.5–11.2
Maize bran	45.5 (25)	32.0 (8)	<1.8–33.2	5.6 *	2.9–12.1	9.7 ± 9.2	6.1–13.4
Pollard	25.5 (14)	0.0 (0)	<1.8–8.9	2.8	0.9–3.8	3.2 ± 2.5	1.9–4.5
Rice bran	5.5 (3)	0.0 (0)	<1.8–4.5	4.0	0.9–4.5	3.1 ± 2.0	0.8–5.4
Maize germ	1.8 (1)	0.0 (0)	4.0	4.0	-	4.0	-
**Animal proteins**							
Dried silver cyprinid fish	16.4 (9)	11.1 (1)	2.34–13.4	3.4	2.8–4.7	4.4 ± 3.4	2.1–6.7
Fish meal	16.4 (9)	33.3 (3)	3.18–29.1	7.0 *	3.6–21.3	12.4 ± 9.5	6.1–18.7
Fresh water shrimp	3.6 (2)	0.0 (0)	<1.8–2.7	1.8	0.9–2.7	1.8 ± 1.3	−0.1–3.7
Bone meal	3.6 (2)	50.0 (1)	9.5–21.6	3.8	2.9–5.4	10.5 ± 1.4	8.5–12.5
Blood meal	1.8 (1)	0.0 (0)	4.0	4.0	-	4.0	-
**Cereal grains**							
Wheat	9.1 (5)	40.0 (2)	2.19–13.4	2.6	2.2–13.4	6.5 ± 5.7	1.4–11.6
Maize	1.8 (1)	0.0 (0)	3.2	3.2	-	3.2	-
Rice	1.8 (1)	0.0 (0)	8.9	8.9	-	8.9	-
**Others**							
Greens	11.0 (6)	16.7 (1)	<1.8–13.4	2.5	0.9–12.3	3.8 ± 4.8	−0.1–7.7
Multivitamin	10.3 (6)	16.7 (1)	2.40–13.4	3.8	2.5–12.4	5.1 ± 4.1	1.7–8.4
Dairy meal	5.5 (3)	0.0 (0)	<1.8–4.0	2.2	0.9–4.0	2.3 ± 1.6	0.6–4.1
Poultry manure	5.5 (3)	0.0 (0)	<1.8–2.0	0.9	0.9–2.0	1.2 ± 0.6	0.5–2.0

Key: ML, maximum level; CI, confidence interval; SD, standard deviation; ≥, greater than or equal to; <, less than; µg/kg, micrograms per kilogram. * Significant difference between feeds with ingredient and those without ingredient where *p* ≤ 0.05.

**Table 6:** Fish health problems reported in farms visited.

Reported Health Problems	Rainbow Trout (*n* = 8) % (*n*)	Tilapia (*n* = 14) % (*n*)	Total (*n* = 22) % (*n*)	*p* Value
No mortalities	75.0 (6)	64.3 (9)	68.2 (15)	1.000
Mortalities	25.0 (2)	35.7 (5)	31.8 (7)	
Normal appetite	87.5 (7)	92.9 (13)	90.9 (20)	1.000
Poor appetite	12.5 (1)	7.1 (1)	9.1 (2)	
Normal growth rates	75.0 (6)	42.9 (6)	54.5 (12)	0.204
Poor growth rates	25.0 (2)	57.1 (8)	45.5 (10)	
No tumor-like lesions	12.5 (1)	92.9 (13)	63.6 (14)	<0.001 *
Tumor-like lesions	87.5 (7)	7.1 (1)	36.4 (8)	

Key: * Significnat difference where *p* ≤ 0.05.

**Table 7:** Pathological lesions observed on gross and microscopic examination of fish.

Pathological Lesions Observed		Rainbow Trout		Tilapia		Total	
		Farms (*n* = 8)	Fish (*n* = 80)	Farms (*n* = 4)	Fish (*n* = 40)	Farms (*n* = 12)	Fish (*n* = 120)
		% (*n*)	% (*n*)	% (*n*)	% (*n*)	% (*n*)	% (*n*)
**Gross lesions**							
	Swollen abdomen	62.5 (5)	46.3 (37)	50.0 (2)	12.5 (5)	58.3 (7)	35.0 (42)
	Enlarged liver	62.5 (5)	60.0 (48)	0.0 (0)	0.0 (0)	41.7 (5)	40.0 (48)
	Nodules or cystic swellings in liver	62.5 (5)	50.0 (40)	0.0 (0)	0.0 (0)	41.7 (5)	33.3 (40)
	Liver hemorrhages	100.0 (8)	91.3 (73)	25.0 (1)	17.5 (7)	75.0 (9)	66.7 (80)
	Muscular hemorrhages	50.0 (4)	30.0 (24)	0.0 (0)	0.0 (0)	33.3 (4)	20.0 (24)
	Enlarged heart	62.5 (5)	40.0 (32)	0.0 (0)	0.0 (0)	41.7 (5)	26.7 (32)
	Enlarged kidneys	62.5 (5)	56.3 (45)	0.0 (0)	0.0 (0)	41.7 (5)	37.5 (45)
	Hemorrhagic intestinal content	37.5 (3)	32.5 (26)	50.0 (2)	17.5 (7)	41.7 (5)	27.5 (33)
	Total gross lesions (*n* = 8)	100.0 (8)	50.8 (325)	37.5 (3)	5.9 (19)	100.0 (8)	35.8 (344)
**Microscopic lesions in liver**							
	Irregular hepatic cords	62.5 (5)	57.5 (46)	0.0 (0)	0.0 (0)	41.7 (5)	38.3 (46)
	Abnormal hepatocytes	62.5 (5)	62.5 (50)	25.0 (1)	7.5 (3)	50.0 (6)	44.2 (53)
	Liver necrosis	87.5 (7)	61.3 (49)	25.0 (1)	20.0 (8)	66.7 (8)	47.5 (57)
	Cytoplasmic vacuoles in hepatocytes	100.0 (8)	100.0 (80)	0.0 (0)	0.0 (0)	66.7 (8)	66.7 (80)
	Hyperchromatic nucleus	87.5 (7)	72.5 (58)	0.0 (0)	0.0 (0)	58.3 (7)	48.3 (58)
	Prominent nucleolus	75.0 (6)	45.0 (36)	0.0 (0)	0.0 (0)	50.0 (6)	30.0 (36)
	Total microscopic lesions (*n* = 6)	100.0 (6)	66.5 (319)	33.3 (2)	4.6 (11)	100.0 (6)	45.8 (330)
Total pathological lesions (*n* = 14)		100.0 (14)	57.5 (644)	33.3 (2)	5.4 (30)	100.0 (14)	40.1 (674)
